# A Systematic Review on the Existing Screening Pathways for Lynch Syndrome Identification

**DOI:** 10.3389/fpubh.2017.00243

**Published:** 2017-09-12

**Authors:** Alessia Tognetto, Maria Benedetta Michelazzo, Giovanna Elisa Calabró, Brigid Unim, Marco Di Marco, Walter Ricciardi, Roberta Pastorino, Stefania Boccia

**Affiliations:** ^1^Section of Hygiene, Institute of Public Health, Università Cattolica del Sacro Cuore, Rome, Italy; ^2^Department of Public Health and Infectious Diseases, Sapienza University of Rome, Italy; ^3^Section of Hygiene, Institute of Public Health, Università Cattolica del Sacro Cuore, Fondazione Policlinico “A. Gemelli”, Rome, Italy; ^4^Italian National Institute of Health (Istituto Superiore di Sanita—ISS), Rome, Italy

**Keywords:** colorectal cancer, hereditary colorectal cancer, Lynch syndrome, immunohistochemistry, microsatellite instability, mismatch repair genes, cancer prevention, screening pathways

## Abstract

**Background:**

Lynch syndrome (LS) is the most common hereditary colon cancer syndrome, accounting for 3–5% of colorectal cancer (CRC) cases, and it is associated with the development of other cancers. Early detection of individuals with LS is relevant, since they can take advantage of life-saving intensive care surveillance. The debate regarding the best screening policy, however, is far from being concluded. This prompted us to conduct a systematic review of the existing screening pathways for LS.

**Methods:**

We performed a systematic search of MEDLINE, ISI Web of Science, and SCOPUS online databases for the existing screening pathways for LS. The eligibility criteria for inclusion in this review required that the studies evaluated a structured and permanent screening pathway for the identification of LS carriers. The effectiveness of the pathways was analyzed in terms of LS detection rate.

**Results:**

We identified five eligible studies. All the LS screening pathways started from CRC cases, of which three followed a universal screening approach. Concerning the laboratory procedures, the pathways used immunohistochemistry and/or microsatellite instability testing. If the responses of the tests indicated a risk for LS, the genetic counseling, performed by a geneticist or a genetic counselor, was mandatory to undergo DNA genetic testing. The overall LS detection rate ranged from 0 to 5.2%.

**Conclusion:**

This systematic review reported different existing pathways for the identification of LS patients. Although current clinical guidelines suggest to test all the CRC cases to identify LS cases, the actual implementation of pathways for LS identification has not been realized. Large-scale screening programs for LS have the potential to reduce morbidity and mortality for CRC, but coordinated efforts in educating all key stakeholders and addressing public needs are still required.

## Introduction

Colorectal cancer (CRC) is the third major cause of cancer-related deaths throughout the world, accounting for 774,000 deaths in 2015 (1). The estimated incidence of CRC is 1.4 million new cases per year worldwide and, of which, approximately 3–5% related to Lynch syndrome (LS) (2, 3).

Lynch syndrome, also known as hereditary non-polyposis colorectal cancer (HNPCC), confers a higher risk of developing CRC (25–70%) and other tumors, including endometrial and ovarian carcinoma as the most common ones (4). LS is an autosomal dominant disorder associated with mutations in the mismatch repair (MMR) genes (*MLH1, MSH2, MSH6*, and *PMS2*,) or in the *EPCAM* gene (4–9). Defective MMR system results in an increased rate of DNA replication errors, causing a microsatellite instability (MSI). MSI phenotype can be detected through polymerase chain reaction (PCR) biomolecular test, while the lack of expression of MMR proteins can be identified using immunohistochemical methods.

Microsatellite instability, although sensitive, is not specific for LS-associated cancers, as approximately 15% of sporadic CRCs also demonstrate MSI (10). Sporadic MSI-high CRCs are typically characterized by aberrant patterns of DNA methylation and by mutations in the *BRAF* gene. Therefore, the *BRAF* mutation and *MLH1* methylation tests are used to distinguish sporadic from LS-associated CRC (11).

While the scientific knowledge about LS is increasing, the question about how LS-induced CRC can be detected and prevented is still an open issue.

Traditionally, the risk assessment for LS was performed through clinical criteria such as the Amsterdam Criteria or the Bethesda Guidelines (12, 13). However, clinical criteria have been criticized for being too complex and lacking in specificity and sensitivity (5). In 2009, the Evaluation of Genomic Applications in Practice and Prevention (EGAPP) working group recommended that all CRCs were screened for LS using either immunohistochemistry (IHC) or MSI testing (14). A positive screening test is followed by genetic counseling and DNA test for MMR mutations to confirm LS diagnosis. However, the potential clinical and economic impact of limiting tumor-based LS screening to individuals below a certain age cutoff was debated (5, 15).

On February 2017, the National Institute for Health and Care Excellence (NICE) confirmed the universal screening approach recommending to offer testing to all newly diagnosed patients with CRC, again, through MSI testing or IHC, and to guide further sequential testing for LS (16).

Identification of LS gives the mutation carriers the possibility to reduce cancer risk through intensive screening and follow-up programs and prophylactic surgery (17, 18).

Despite the great interest in this field of research, the identification of individuals who should undergo LS genetic testing is a critical issue and the practice about LS screening remains very heterogeneous. This prompted us to conduct a systematic review of the existing screening pathways for LS after the EGAPP recommendations were released.

## Materials and Methods

### Search Strategy

We identified the studies through a search of MEDLINE, ISI Web of Science, and SCOPUS online databases.

The search has been limited to articles published in English language from January 1, 2010, until November 7, 2016. We used the following terms for the literature search: (“genetic services” OR “genetic service provision” OR “genetic service delivery” OR “genomic service delivery” OR “genetic delivery models” OR pathway OR screening) AND (“Lynch syndrome” OR “hereditary non-polyposis colorectal cancer”).

Three investigators (Brigid Unim, Alessia Tognetto and Maria Benedetta Michelazzo) independently reviewed titles, abstracts, and full texts of the retrieved papers in order to identify the eligible studies. Results were cross-checked and any disagreement was resolved through discussion until consensus was reached. The snowball strategy, a manual search of the references reported by studies retrieved from the online databases, was also adopted to identify additional studies. The systematic review was drafted in accordance with PRISMA guidelines (19).

### Eligibility Criteria

Studies that reported on structured and permanent screening pathways for the identification of LS carriers were deemed as eligible. We excluded studies that analyzed pilot pathways. When the same subjects were included in several publications, we included the most recent report.

### Data Extraction

We extracted the name of the first author, the year of publication, the country and the setting where the screening pathway was activated, and the time period when the screening pathway was evaluated. For each screening pathway, the following data were extracted: inclusion criteria for screening, screening pathway and methodology, health-care professionals involved, number of patients screened during the evaluation period, and indicators of the effectiveness of the pathway in terms of LS carriers detected. Detection rate was reported as percentages, if provided in the study, or calculated as percentage dividing the number of LS carriers by the number of patients screened (if the data were available).

## Results

### Study Selection

**Figure 1:**
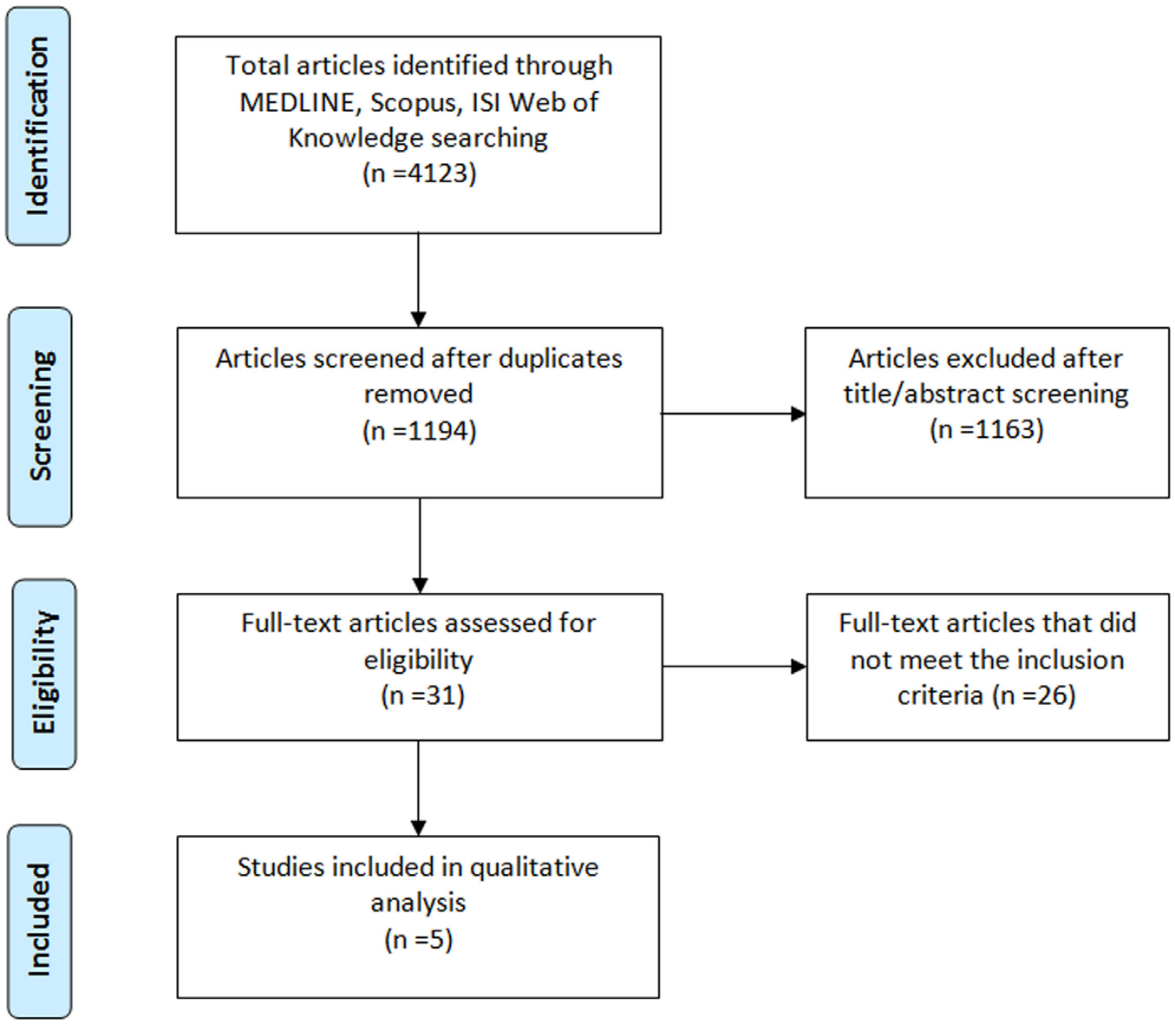
Flowchart of the literature selection.

Our search yielded 4,123 records in the initial screening phase. After removing duplicates, we screened a total of 1,194 articles. Among them, 1,163 were excluded because unrelated to the research topic after title and abstract screening. The remaining 31 full-text articles were assessed for eligibility, and 26 were further excluded as they did not meet the inclusion criteria. The total number of studies included in the qualitative analysis was 5 (20–24). Figure 1 shows the study selection process and the results of the literature search.

### Study Characteristics

The main characteristics of the included studies are reported in Tables 1 and 2.

**Table 1:** Characteristics of the included studies (*N* = 5).

Reference	Country	Setting	Time period
Heald et al. (20)	Ohio (USA)	Cleveland Clinic	2009–2012 (42 months)
Schofield et al. (21)	West Australia	PathWest Laboratory Medicine WA, St. John of God Pathology, Western Diagnostic Pathology, Genetic Services WA	2008–2012 (60 months)
Kidambi et al. (22)	California (USA)	San Francisco General Hospital	2009–2014 (72 months)
Cohen et al. (23)	Washington (USA)	Seattle Cancer Care Alliance, Washington University Medical Center	2013 (6 months)
Zumstein et al. (24)	Switzerland (Europe)	St. Claraspital Hospital	2011–2015 (52 months)

**Table 2:** LS screening pathways.

Country	Inclusion criteria	Screening pathway and methodologies	Healthcare professionals involved	Number of patients screened	LS carriers detected	LS detection rate (%)
Ohio (USA)	Universal CRC screening	(1) MSI test or IHC (2) *BRAF* test if *MLH1* loss	Pathologist	784	17	2.2
		(3) Genetic counseling recommendation	Colorectal surgeon			
		(4) Genetic counseling and germline testing	Genetic counselor			
West Australia	CRC with any of the following: ➢ <60 years ➢ Individual or family history of cancer	(1) IHC (2) MSI test for confirmation (3) *BRAF* test if MSI^+^ and *MLH1* loss and/or *PMS2* expression	Pathologist	NR	42	NR
		(4) Genetic counseling recommendation	Treating clinician			
	➢ Histological characteristics	(5) Genetic counseling and germline testing	NR			
California (USA)	CRC with any of the following: ➢ ≤50 years	(1) IHC (2) *BRAF* test if *MLH1* loss	Pathologist	57	3	5.3
	➢ Histological characteristics	(3) Genetic counseling recommendation	Treating clinician or multidisciplinary team			
	➢ Synchronous CRC	(4) Genetic counseling and germline testing	Genetic counselor			
Washington (USA)	Universal CRC screening	(1) MSI test or IHC, or MSI test + IHC (2) *BRAF* test if *MLH1* loss or MSI-high	Pathologist	31	0	0
		(3) Genetic counseling recommendation	Multidisciplinary team			
		(4) Genetic counseling and germline testing	Genetic counselor			
Switzerland	Universal CRC screening	(1) IHC (2) *BRAF* test (or IHC) if *MLH1* loss	Pathologist	486	4	0.8
		(3) Genetic counseling recommendation	Multidisciplinary team			
		(4) Genetic counseling and germline testing	Geneticist			

*NR, not reported; CRC, colorectal cancer; IHC, immunohistochemistry; LS, Lynch syndrome; MSI, microsatellite instability*.

Of the five studies included in the review, one was conducted in West Australia, three in the USA (California, Ohio, and Washington), and one in Switzerland.

All studies described a structured and permanent screening pathway for the identification of LS carriers and analyzed the effectiveness of the pathways in the period ranging from 2008 to 2015. The median duration of the evaluation was 52 months, with a minimum value of 6 months (23), and a maximum value of 72 months (22) (Table 1).

### LS Screening Pathways Description

All the LS screening pathways described in the literature started from CRC cases and did not take into consideration cascade testing for healthy family members of the index cases (Table 2).

Regarding the inclusion criteria for screening, three pathways (20, 23, 24) included all newly diagnosed patients with CRC (universal tumor screening). Two pathways included only those CRC patients who met specific criteria: age below a certain cutoff and personal/familiar history of cancer, or histological characteristics of the CRC suggestive for LS (21, 22).

Concerning the laboratory tests used, three pathways (20, 21, 23) used IHC and/or MSI testing, while in the California’s (22) and Switzerland’s (24) pathways only IHC was performed. In case of *MLH1* loss, a PCR test was performed to assess the somatic mutation V600E in *BRAF* gene, in all the screening pathways; in the Switzerland’s pathway (24) this could be assessed also, or alternatively, with IHC.

Since these tests are performed in laboratories, in all the aforementioned pathways the health-care professionals involved were pathologists. If the results of the tests indicated a risk for LS syndrome, the decision if a referral to a genetic specialist is appropriate involved a multidisciplinary team in Washington’s and Switzerland’s pathways (23, 24), while in Ohio and West Australia’s ones (20, 21), it involved the treating clinician. In the California’s pathway (22), instead, patients were identified as candidates for genetic counseling either by a multidisciplinary team or by the treating clinician.

In all the pathways, the genetic counseling, performed by a geneticist or a genetic counselor, was mandatory to undergo DNA genetic testing.

### Outcome Results

According to the available data, the pathway with the largest cohort analyzed was the Ohio’s pathway with 784 patients screened (20), followed by the Switzerland’s pathway with 486 patients (24). The Washington’s pathway and the California’s pathway screened 31 and 57 patients, respectively (22, 23).

The overall LS detection rate in the evaluation period ranged from 0% in Washington’s pathway to 5.3% in California’s pathway (23, 24) (Table 2).

## Discussion

This review aimed to describe the existing screening pathways for the identification of LS carriers. We performed a systematic literature search and we retrieved five experiences of structured and permanent screening pathways, of which three were performed in the USA, one in Australia, and one in Europe (20–24).

All the studies retrieved were published after the publication of the EGAPP recommendations that endorsed screening of all newly diagnosed CRC individuals (regardless of age or family history) (14). Afterward, this strategy has been strongly recommended by the American Gastroenterological Association Institute and by the US Multi-Society Task Force on Colorectal Cancer, as well as by European experts (25–27). On February 2017, the NICE confirmed further the universal tumor screening approach (16).

Concerning the inclusion criteria, three of the five pathways followed a universal tumor screening approach, that starts from all newly diagnosed patients with CRC (20, 23, 24). The remaining two pathways included CRC patients with specific criteria. Namely, the Australian pathway included CRC patients aged <60 years, with a personal/familiar history of cancer, or with histological characteristics of the CRC suggestive for LS (21). The Californian pathway, instead, included CRC patients ≤ 50 years, that had tumors with histological features suggestive of MSI, or that had synchronous CRC (22). In all the studies, laboratory procedures included MSI testing and/or IHC to identify tumor MMR anomalies. On a subset of MMR-deficient tumors, the addition of the *BRAF* V600E mutation analysis can help in finding out sporadic forms.

The effectiveness of the pathways is variable in terms of LS detection rate, and this could be partly explained by the different cohort sizes, length of the evaluation period, and the peculiar organization of the health-care systems.

Although universal CRC tumor screening is theoretically feasible, its implementation is demanding and the potential clinical and economic impact of limiting tumor-based LS screening to individuals below a certain age cutoff is debated. The age limit selected can be a compromise between the costs of tests and surveillance measures for high-risk individuals and the probability of identifying the most number of LS carriers (28). Furthermore, in resource-limited settings, screening only red flag cases could be more easily achievable, also considering a disparity among different types of health-care systems (29).

Universal tumor screening has been proved to be also cost-effective, because it allows reduction of the costs related to morbidity and mortality from CRC from the early identification of LS carriers among family members (15). The five experiences, however, did not take into consideration cascade testing for family members of the index cases. Early detection of individuals with LS and their relatives is relevant since they can follow intensive cancer surveillance programs, which may reduce morbidity and mortality (17, 18). Surveillance recommendations for LS carriers are different from those of the general population, because premalignant colorectal adenoma tends to turn more rapidly into carcinoma and the increased incidence of extracolonic cancers can be prevented through prophylactic surgery (18, 30, 31).

Therefore, epidemiological data show that systematics, long-term aspirin use reduced incidence and mortality due to CRC (32). The highest influence of chemopreventive strategies is expected in patients with defined hereditary predisposition as LS (33, 34), also considering the estimates of the LS-prevalence in the general population (35, 36).

However, the favorable outcome of this process is dependent on patients receiving the screening results with consequent pursuit of genetic counseling and genetic testing.

The studies retrieved reported a lower compliance with genetic counseling referral than the expected, and the main reasons were loss of patients during different phases of the screening pathway, lack of communication between patient and physicians, and a refusal of genetic counseling by the patients due to a perceived lack of benefit (20–24). These findings highlighted an effective educational need for patients, and a request of cooperation and effective communication between the health-care professionals. Several studies have reported different strategies to reduce barriers and increase compliance with follow-up genetic counseling and testing, with variable outcome (37–39). As described in three of the five pathways, a multidisciplinary team is often involved in the screening pathways, therefore, a standardized plan must be created with roles and responsibilities clearly assigned.

Some limitations of this study need to be considered when interpreting the findings. First, the inclusion criteria required that the studies analyzed a structured and permanent screening pathway for the identification of LS carriers. Therefore, our review summarized the procedure for LS detection in routine clinical practice. Hence, we found a rather small number of studies in line with our criteria, and this has clearly led to poor generalizability of the models of identification currently experienced. Furthermore, as in all systematic reviews, publication bias might be an issue, so that we might have lost few small unpublished studies.

Nonetheless, this study represents the first systematic review trying to identify and describe the worldwide experiences of different LS screening pathways in different clinical settings. The practice about LS screening appears very heterogeneous in terms of inclusion criteria, laboratory procedures, and health-care professionals involved.

In conclusion, this review aimed to cover the significant gap on how health-care organizations currently screen for LS. The results show that only a small number of experiences about structured tumor screening programs for LS have been reported in the literature and the cascade testing for family members of the index cases is not currently implemented.

The importance of LS identification is one of the priority topics, as stressed also in one of the agenda items in Healthy People 2020: “Increase the proportion of persons with newly diagnosed colorectal cancer who receive genetic testing to identify Lynch syndrome” (40–41). The advantages of large-scale screening in terms of efficacy, quality, and costs are documented in research, but coordinated efforts in educating all key stakeholders and addressing public needs are necessary to translate evidence-based recommendations into health gain.

## Author Contributions

SB conceived the study and RP, AT, and MM participated in its design. BU, MM, and AT identified the studies through a search of MEDLINE, ISI Web of Science, and SCOPUS online databases and performed the data extraction from the papers. SB, RP supervised AT, MM, and BU. SB, WR, RP, AT, GEC, MM critically discussed and interpreted the results of the review. All the authors drafted and critically reviewed this manuscript and approved the final version.

## Conflict of Interest Statement

The authors declare that the research was conducted in the absence of any commercial or financial relationships that could be construed as a potential conflict of interest.
